# Celiac disease as a potential cause of idiopathic portal hypertension: a case report

**DOI:** 10.1186/1752-1947-3-68

**Published:** 2009-02-16

**Authors:** Farhad Zamani, Afsaneh Amiri, Ramin Shakeri, Ali Zare, Mehdi Mohamadnejad

**Affiliations:** 1 Gastrointestinal and Liver Disease Research Center, Firouzgar Hospital, Iran University of Medical Sciences, Tehran, Iran; 2 Digestive Disease Research Center, Shariati Hospital, Tehran University of Medical Sciences, Tehran, Iran; 3 Department of Pathology, Firouzgar Hospital, Iran University of Medical Sciences, Tehran, Iran

## Abstract

**Introduction:**

Idiopathic portal hypertension is a disorder of unknown etiology, clinically characterized by portal hypertension, splenomegaly and anemia secondary to hypersplenism.

**Case presentation:**

A 54-year-old man was admitted to our hospital for evaluation of malaise, weight loss, abdominal swelling and lower limb edema. His paraclinical tests revealed pancytopenia, large ascites, splenomegaly and esophageal varices consistent with portal hypertension. Duodenal biopsy and serologic findings were compatible with celiac disease. His symptoms improved on a gluten-free diet, but his clinical course was further complicated with ulcerative jejunoileitis, and intestinal T-cell lymphoma.

**Conclusion:**

It seems that celiac disease, by an increased immune reaction in the splenoportal axis, can result in the development of idiopathic portal hypertension in susceptible affected patients.

## Introduction

Idiopathic portal hypertension (IPH) is a disorder generally classified as a noncirrhotic portal hypertension of unknown etiology, and is clinically characterized by portal hypertension, splenomegaly and pancytopenia [1].

In some cases, IPH may be related to autoimmune reactions and immunologic abnormalities [2]. On the other hand, celiac disease (CD) is an immune-mediated enteropathy due to the ingestion of a gluten containing diet. It has been suggested that in CD the deposition of circulating immune complexes originating from the small bowel may cause other diseases [3]. The association of CD with IPH has been recently reported in the literature [4-6].

Here we report on a patient with celiac disease complicated by idiopathic portal hypertension, whose symptoms and signs of portal hypertension improved on a gluten free diet (GFD). However, the patient's clinical course was further complicated with ulcerative jejunoileitis and intestinal T-cell lymphoma.

## Case presentation

A 54-year-old Iranian man was admitted to our hospital in May 2006 because of malaise, weight loss and edema of the lower limbs beginning 2 months prior to admission. He also had a history of iron-deficient anemia and increasing abdominal swelling for 8 months prior to admission. On physical examination he was cachectic and pale in appearance, with normal vital signs. The conjunctiva was pale. Chest and heart were normal. On abdominal examination, tense ascites was detected. There was also a 2+ pitting edema of the lower limbs. His initial lab tests were normal except for anemia, thrombocytopenia and leucopenia (Table 1).

**Table 1:** Results of laboratory tests

Variable	Normal value	First admission	3 months after GFD
Hemoglobin (g/dL)	13.5–17.5	7.2	11.1
White-cell count (per mm^3^)	4,500–11,000	1,800	8,900
Platelet count (per mm^3^)	150,000–450,000	86,000	128,000
MCV (fl)	77–97	68	81
MCH (pg/cell)	26–32	26	28
Serum Albumin (g/dL)	3.5–5.5	3.1	3.1
PT (Seconds)	11–13	13	13
PTT (Seconds)	26–38	30	31
AST (U/L)	5–40	28	28
ALT (U/L)	5–40	34	32
Total Bilirubin (mg/dl)	0.2–1.2	0.5	0.5
Alkaline phosphatase (U/L)	64–306	203	202
BUN (mg/dL)	5–17	17	15
Creatinine(mg/dL)	0.5–1.4	1.1	1

Abbreviations: GFD, gluten free diet; MCV, mean corpuscular volume; MCH, mean corpuscular hemoglobin; PT, prothrombin time; PTT, partial thromboplastin time; AST, aspartate aminotransferase; ALT, alanine aminotransferase; BUN, blood urea nitrogen.

Abdominal sonography revealed splenomegaly (23 × 7 cm) and large amounts of ascites. Portal vein diameter was 18^mm ^with a blood velocity of 25 cm/s. Duplex doppler ultrasonography of the splanchnic venous system was consistent with portal hypertension. Ultrasound showed no evidence of vascular obstruction in the splenoportal axis. On CT scan, there was no lymph node enlargement compressing the portal splenic axis.

**Figure 1:**
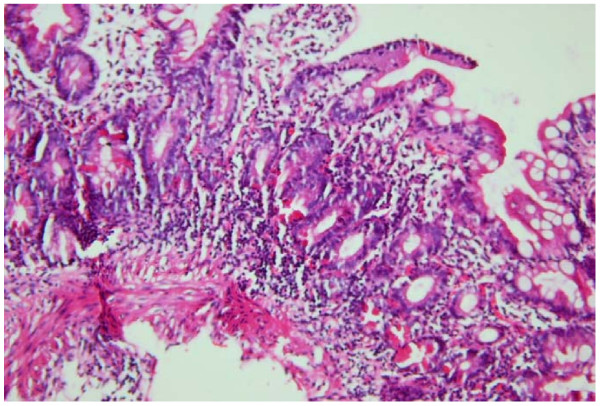
**Microscopic image of the duodenal mucosal biopsy**. Duodenal mucosal biopsy showing subtotal villous atrophy, lymphocyte infiltration and crypt hyperplasia.

Eosophagogastroduodenoscopy showed four columns of grade three esophageal varices with red signs, small gastric fundal varices and moderate portal hypertensive gastropathy. The duodenal bulb was normal but the second part of the duodenum had atrophic folds and scalloping. Results of duodenal biopsy revealed lymphocyte infiltration, crypt hyperplasia and villous atrophy compatible with CD, grade Шb according to the Marsh classification [7] (Figure 1). Serologic studies revealed positive anti tissue transglutaminase (tTG) and anti endomysial antibody (EMA). Percutaneous liver biopsy revealed mild non-specific chronic hepatitis.

Liver function tests including serum albumin and prothrombin time were normal. Hepatitis B surface antigen, hepatitis C antibody, and human immunodeficiency virus antibody were negative.

Serologies were not consistent with autoimmune hepatitis, or primary biliary cirrhosis. Anti-nuclear antibody (ANA), anti-smooth muscle antibody (ASMA), antiobodies to anti-liver/kidney microsomes (ALKM-1), antimitochondrial antibody (AMA), and anti-liver cytosol antigen antibody (ALC-1), were negative. The serum gammaglobulin level was normal.

A gluten free diet was advised. At a 3 month follow-up visit, the patient had gained 3 kg; and his hemoglobin, platelet count and white blood cell count were increased (Table 1). On physical examination there was mild peripheral edema, and only a small amount of ascites.

Furthermore, his spleen size decreased to 17 × 6 cm by ultrasonography. He did not return for follow-up visits until 1 year later, when he was admitted again to our hospital because of progressive malaise, anorexia, diarrhea, abdominal pain and weight loss. He had not been compliant with a GFD in the past year despite our recommendation. Abdominal CT scan showed splenomegaly, thickening of the small intestine, and multiple soft tissue densities in the mesentery. Push enteroscopy revealed multiple jejunal ulcers and a mass lesion, which was biopsied. Seven days later the patient developed an acute abdomen and underwent an emergency laparotomy. Perforations of the small intestine at two sites, 100 and 135 cm from the ligament of Treitz, were seen. Also, during laparotomy, the liver did not look cirrhotic and biopsies were compatible with ulcerative jejunitis and intestinal T-cell lymphoma. The patient was referred to an oncologist but unfortunately died 2 weeks later.

## Discussion

IPH is a heterogeneous and multifactorial disorder with a potential genetic contribution, seen most often in the Indian subcontinent and in East Asia [8-10]. Trace element chemical theory, autoimmunity theory and infection theory have been suggested in the literature, although none has been clearly proven [11]. The diagnosis of IPH is established by the presence of unequivocal portal hypertension (presence of esophageal varices on endoscopy, increased splenic pressure and collaterals on splenoportovenography (SPV) or ultrasound, by a definite exclusion of cirrhosis and by exclusion of obstruction of the splenoportal axis on SPV and on Doppler ultrasound [12]. In our patient cirrhosis was ruled out by liver biopsy. His liver function tests were totally normal. CD was suggested as a cause of IPH in this patient, as his symptoms improved transiently while he adhered to a GFD. The association of celiac disease with IPH has been recently reported in the literature. The improvement of portal hypertension with a gluten free diet, a rare entity reported in a case, implicates a causal relationship between portal hypertension and increased inflammatory reactions in celiac disease [5]. In celiac disease, autoantibody reactivity to transglutaminase 2 (tTG2) has been shown to closely correlate with the acute phase of the disease. Immune reactivity to other autoantigens, including transglutaminase 3, actin, ganglioside, collagen, calreticulin and zonulin, among others, has also been reported in celiac disease. Some immunologic abnormalities may be associated with specific clinical presentations or extra-intestinal manifestations of celiac disease. There are several reports on the association of CD with different diseases, most with immunologic pathogenesis. Cellular immunity is important, with increased CD8 T lymphocytes and inflammatory cytokines (notably INFγ and IL-10) found in involved tissues [13,14]. IPH may represent aberrant immune activity, triggered by exposure to gluten in CD. The coexistence of CD and IPH suggests that there may be an immunological link between these two conditions. Therefore, testing for CD in patients with IPH is warranted.

In this case report we emphasize two other points. First, clinical deterioration in terms of gastrointestinal symptoms such as diarrhea and abdominal pain should increase the clinical suspicion to the development of ulcerative jejunitis and enteropathy-associated T-cell lymphoma (EALT). Second, our patient had a long history of iron deficiency anemia (IDA) which was neglected at the primary care level. IDA is the most common feature of CD and can be the sole presentation of this disease [15]. The diagnosis of CD in such patients may be delayed or missed, leading to future complications. Careful attention for atypical presentations of CD may allow early diagnosis and prevention of complications.

## Conclusion

Celiac disease may play a role as a trigger for the development of idiopathic portal hypertension. Patients with IPH should be evaluated for CD.

## Abbreviations

IPH: idiopathic portal hypertension; CD: celiac disease; GFD: gluten free diet; EATL: enteropathy-associated T-cell lymphoma; IDA: iron deficiency anemia.

## Competing interests

The authors declare that they have no competing interests.

## Authors' contributions

FZ conceived the study and participated in its design. AA participated in the design of the study, the acquisition and interpretation of data and drafted the manuscript. RS revised the paper. AZ participated in the conception of the study and its design. MM revised the manuscript and gave final approval of the version to be published. All authors contributed intellectual content and have read and approved the final manuscript.

## Consent

The authors state that written informed consent was obtained from the patient's brother for publication of this case report and accompanying image. A copy of the written consent is available for review by the Editor-in-Chief of this journal.
